# Sensitive Spectrophotometric Determination of Atenolol in Pharmaceutical Formulations Using Bromate-Bromide Mixture as an Eco-Friendly Brominating Agent

**DOI:** 10.1155/2012/810156

**Published:** 2012-03-20

**Authors:** Kudige N. Prashanth, Kanakapura Basavaiah

**Affiliations:** Department of Chemistry, University of Mysore, Manasagangotri, Mysore 570006, Karnataka, India

## Abstract

Three simple and sensitive spectrophotometric methods are proposed for the determination of atenolol (ATN) in bulk drug and tablets. The methods are based on the bromination of ATN by the bromine generated *in situ* by the action of the acid on the bromate–bromide mixture followed by the determination of unreacted bromine by reacting with a fixed amount of either meta-cresol purple (MCP) and measuring the absorbance at 540 nm (method A) and 445 nm (method B) or erioglaucine (EGC) and measuring the absorbance at 630 nm (method C). Beer's law is valid within the concentration ranges of 1.0–20.0, 2.0–40.0 and 1.0–8.0 *μ*g/mL for method A, method B and method C, respectively. The calculated molar absorptivities were found to be 1.20×10^4^, 4.51×10^3^ and 3.46 × 10^4^  L/mol · cm for method A, method B and method C, respectively. Sandell's sensitivity values, correlation coefficients, limits of detection and quantification are also reported. Recovery results were statistically compared with those of a reference method by applying Student's *t*- and *F*-test. The novelty of the present study is the measurement of two different colors using MCP, that is, red-pink color of MCP in acid medium at 540 nm and yellowish-orange color of brominated MCP at 445 nm.

## 1. Introduction

Atenolol (ATN), chemically known as 4-(2-hydroxy-3-[(1-methylethyl) amino] propoxy) benzeneacetamide [1], is a *β*1-selective (cardio selective) adrenoreceptor antagonist drug used for antiangina treatment to relieve symptoms, improve tolerance, and as an antiarrhythmic to help regulate heartbeat and infections. It is also used in management of alcohol withdrawal, in anxiety states, migraine prophylaxis, hyperthyroidism, and tremors [2]. The drug is official in Indian Pharmacopoeia [3] which describes a UV-spectrophotometric method and also in British Pharmacopoeia [4] which recommends high-performance liquid chromatographic (HPLC) method for its determination. Several methods have been reported for the determination of ATN in pharmaceutical dosage forms and include diffuse reflectance spectroscopy [5], HPLC [6–26], high-performance thin-layer chromatographic (HPTLC) [27, 28], ultra performance liquid chromatography (UPLC) [29], gas chromatography (GC) [30, 31], nonsuppressed ion chromatography [32], flourometry [33, 34], differential scanning calorimetry (DSC) and thermogravimetry (TG) [35], electrophoresis, [36–38] voltammetry [39], ion-selective electrode- (ISE-) based potentiometry [40], atomic absorption spectrometry (AAS) [41], UV-spectrophotometry [42–50], visible spectrophotometry [51–62], and titrimetry [60–62].

To the best of our knowledge, there are twelve reports on the use of visible spectrophotometry for the determination of ATN in pharmaceutical formulations. Agrawal et al. [51] have reported a method based on the reaction of ATN with hydroxylamine hydrochloride in NaOH medium followed by the reaction of the resultant hydroxamic acid derivative with FeCl_3_ to give a red-violet ferric hydroxamate complex. Assays based on charge transfer complexation reaction of ATN with chloranilic acid have been reported by Agarwal et al. [52] and Yu et al. [53]. Korany et al. [54] have developed a method based on the treatment of a CHCl_3_ extract of powdered tablets of atenolol with acetaldehyde, a halogenated benzoquinone reagent (chloranil, 2,5-dichlorobenzoquinone, or 2,6-dibromobenzoquinone chlorimine), and propan-2-ol. The slow reaction between ATN and ammonium vanadate in sulphuric acid medium resulted in two kinetic spectrophotometric methods (fixed-concentration method and fixed-time method) [55]. Al-Ghannam and Belal [56] used the reaction between ATN and 4-chloro-7-nitrobenzo-2-oxa-1,3-diazole in borate buffer of pH 8 at the boiling temperature for the kinetic spectrophotometric assay of drug. The method developed by Hiremath et al. [57] is based on the oxidation of atenolol by a known excess of permanganate in alkaline media and determination of unreacted permanganate spectrophotometrically at 526 nm. Bashir et al. [58] have reported a method based on determination of ATN in basic medium, followed by addition of sodium nitroprusside to generate a coloured complex. Basavaiah et al. [59] have reported a method based on the oxidation of ATN by a measured excess of chloramine-T followed by determination of the unreacted oxidant by a charge-transfer complexation reaction involving metol and sulphanilic acid. The assay method based on the oxidation of ATN by a known excess of chloramine-T in acid medium followed by determination of the unreacted oxidant by reacting with a fixed amount of either metanil yellow or indigo carmine have been reported by Basavaiah et al. [60]. A similar method [61] employed bromate-bromide mixture, methyl orange as reagents in acid medium. An acid-base reaction employing phenol red has also been reported by the same authors [62]. However, many of the above methods suffered from one or other disadvantage like poor sensitivity, heating or extraction step, use of organic solvents, use of expensive chemical, and/or complicated experimental setup as can be seen from Table 1.

The aim of this study was to develop three new spectrophotometric methods for the assay of ATN based on bromination of ATN by a green brominating agent (i.e., bromine-generated *in situ*). The methods use bromated-bromide mixture, metacresol purple (MCP), and erioglaucine (EGC) as reagents. The proposed methods are economical compared to the previously reported chromatographic techniques. Moreover, these methods are sensitive, simple, does not involve heating or extraction step, and free from usage of hazardous chemicals. Since inexpensive and easily available chemicals are used, the developed methods evidence low cost per analysis.

## 2. Experimental

### 2.1. Apparatus

 All absorbance measurements were made on a Systronics model 106 digital spectrophotometer (Systronics, Ahmadabad, India) provided with 1 cm matched quartz cells.

### 2.2. Materials and Reagents

 All chemicals and reagents used were of analytical or pharmaceutical grade. Distilled water was used to prepare the solutions.

**(1) Bromate-Bromide Mixture (40, 80, and 18 *μ*g/mL)**

A stock standard bromate-bromide mixture solution equivalent to 500 *μ*g/mL KBrO_3_ was prepared by dissolving accurately weighed 50 mg of KBrO_3_ (S. D. Fine Chem. Ltd., Mumbai, India) and 0.5 g of KBr (Merck, Mumbai, India) in water and diluted to the mark in a 100 mL calibrated flask. The stock solution was diluted appropriately with water to get the working concentrations of 40, 80, and 18 *μ*g/mL KBrO_3_ for use in method A, method B, and method C, respectively.

**(2) MetaCresol Purple Solution (80 and 200 *μ*g/mL)**

A 400 *μ*g/mL stock solution was first prepared by dissolving 40 mg of dye (Loba Chemie, Mumbai, India) in 2 mL of 0.1 N NaOH and diluted to volume with water in a 100 mL calibrated flask. The solution (400 *μ*g/mL) was diluted further with water to get the working concentrations of 80 *μ*g/mL and 200 *μ*g/mL MCP solutions.

**(3) Erioglaucine Solution (300 *μ*g/mL)**

The solution was prepared by dissolving 30 mg of dye (Loba Chemie, Mumbai, India) in water and diluting to the mark with water in a 100 mL calibrated flask.

**(4) Hydrochloric Acid (5 M and 1 M)**

The solutions were prepared by appropriate dilution of concentrated hydrochloric acid (S. D. Fine Chem. Ltd., Mumbai, India. Sp. gr. 1.18) with water.

**(5) Standard ATN Solution**

Pharmaceutical grade atenolol (ATN) certified to be 99.89% pure was gifted by Cipla India Ltd., Mumbai, India, and was used as received without any further purification and analysis. A stock standard solution equivalent to 200 *μ*g/mL ATN was prepared by dissolving accurately weighed 50 mg of pure drug with water in a 250 mL calibrated flask. This stock solution was diluted appropriately with water to get the working concentrations of 40 *μ*g/mL for use in methods A and C, and 80 *μ*g/mL for use in method B.

### 2.3. Assay Procedure

#### 2.3.1. Method A (Measuring MCP in Acid Medium)

 Different aliquots (0.25–5.0 mL) of standard ATN solution (40 *μ*g/mL) were accurately transferred into a series of 10 mL calibrated flasks using microburette and the total volume was adjusted to 5.0 mL by adding requisite volume of water. To each flask, 2 mL of 5 M HCl was added followed by 1 mL of bromate-bromide mixture (40 *μ*g/mL in KBrO_3_). The content was mixed well and the flasks were allowed to stand for 15 min with occasional shaking. Then, 1 mL of 80 *μ*g/mL MCP was added to each flask, diluted to the mark with water, mixed well, and the absorbance of each solution was measured at 540 nm against a reagent blank after 5 min.

#### 2.3.2. Method B (Measuring Brominated Product of MCP)

 Varying aliquots (0.25–5.0 mL) of a standard solution (80 *μ*g/mL ATN) were accurately measured into a series of 10 mL calibrated flasks and the total volume was brought to 5 mL by adding water. To each flask were added 2 mL of 5 M HCl and 1 mL of KBrO_3_-KBr solution (80 *μ*g/mL, in KBrO_3_). The content of each flask was mixed well and kept aside for 10 min with occasional swirling. At last, 1 mL of 200 *μ*g/mL MCP solution was added to each flask and diluted up to the mark with water. The absorbance of each solution was measured after 5 min at 445 nm against water.

#### 2.3.3. Method C (Using EGC)

 Aliquots (0.25–2.0 mL) of a standard ATN (40 *μ*g/mL) solution were accurately transferred into a series of 10 mL calibrated flasks and the total volume was adjusted to 2.0 mL with water. To each flask, 5 mL of 1 M HCl was added followed by 1.0 mL of bromate-bromide mixture (18 *μ*g/mL, in KBrO_3_). The content was mixed and the flasks were let stand for 10 min with occasional shaking followed by addition of 1 mL of 300 *μ*g/mL EGC to each flask. The solutions were diluted to the mark with water, mixed well, and the absorbance of each solution was measured at 630 nm after 5 min against a reagent blank.

#### 2.3.4. Analysis of Commercial Tablets

 Twenty tablets each containing 25, 50, or 100 mg of ATN were weighed accurately and pulverized. An amount of powdered tablet equivalent to 20 mg of ATN was transferred into a 100 mL calibrated flask and 60 mL of water was added. The content was shaken thoroughly for about 15–20 min, diluted to the mark with water, mixed well, and filtered using a Whatman No. 42 filter paper. The first 10 mL portion of the filtrate was discarded and a suitable aliquot of the filtrate (200 *μ*g/mL ATN) was diluted to get the working concentrations of 40 *μ*g/mL ATN for the assay by methods A and C, and 80 *μ*g/mL ATN for method B.

#### 2.3.5. Analysis of Placebo Blank

 A placebo blank of the composition: talc (45 mg), starch (35 mg), acacia (25 mg), methyl cellulose (40 mg), sodium citrate (25 mg), magnesium stearate (35 mg), and sodium alginate (30 mg) was made and its solution was prepared in 25 mL calibration flask as described under Section 2.3.4, and then subjected to analysis using the procedures described above.

#### 2.3.6. Analysis of Synthetic Mixture

 To the placebo blank of the composition described above, 20 mg of ATN was added and homogenized, transferred to a 100 mL calibrated flask, and the solution was prepared as described under Section 2.3.4, and then subjected to analysis by the procedures described above. The analysis was used to study the interferences of excipients such as talc, starch, acacia, methyl cellulose, sodium citrate, magnesium stearate, and sodium alginate.

## 3. Results and Discussion

### 3.1. Absorption Spectra

**Figure 1:**
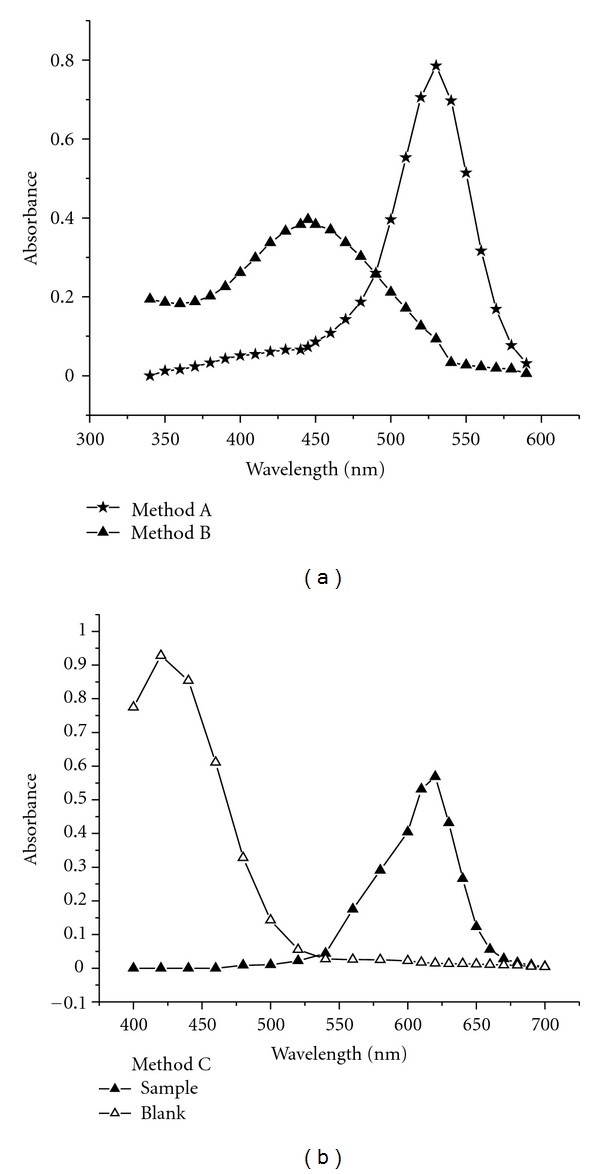
Method A: 1 mL 80 *μ*g/mL MCP; 2 mL 5 M HCl. Method B: Brominated product of MCP (1 mL 40 *μ*g/mL bromated-bromide mixture; 2 mL 5 M HCl; 1 mL 80 *μ*g/mL MCP). Method C: Sample: 1 mL 40 *μ*g/mL ATN; 5 mL 1 M HCl; 1 mL 18 *μ*g/mL bromated-bromide mixture; 1 mL 300 *μ*g/mL EG. Blank: 5 mL 1 M HCl; 1 mL 18 *μ*g/mL bromated-bromide mixture; 1 mL 300 *μ*g/mL EG.

The proposed methods are based on the determination of residual bromine generated *in situ *after the reaction between the drug and bromine is judged to be complete. The red-pink color of unreacted MCP in acid medium was absorbed maximally at 540 nm (method A). The residual bromine was then used to brominate MCP yielding yellow-colored bromo-derivative product with *λ*_max⁡_ at 445 nm (method B). Similar to method A, the green color of unreacted EGC in acid medium peaked at 630 nm (method C). The absorption spectra of all methods are presented in Figure 1.

### 3.2. Chemistry

**Figure 2:**
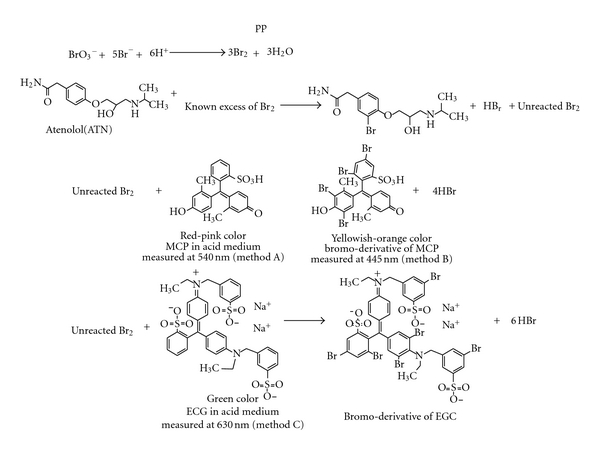
Tentative reaction scheme for the proposed methods.

 Atenolol is reported to undergo bromination by bromine generated *in situ* by the action of the acid on the bromate-bromide mixture [61]. The solution of bromate-bromide mixture in acid medium behaves as an equivalent solution of bromine and has been used for the assay of several pharmaceutical compounds [63–66]. The present investigation deals with three spectrophotometric methods for the assay of ATN using bromine generated *in situ* as eco-friendly brominating agent and avoiding the use of highly toxic and hazardous liquid bromine. The proposed methods are indirect and based on the bromination of ATN by the bromine followed by the determination of unreacted bromine by reacting with a fixed amount of either MCP or EGC and measuring the absorbance at the respective wavelengths. The reaction between ATN and bromine generated *in situ* uses electrophilic substitution reaction at one orthoposition to the alkoxy group on the benzene ring. The unreacted bromine was determined by its reaction with either MCP or EGC. The reaction of bromine with MCP involved two simultaneous processes, that is, decrease in the pink color of MCP in acid medium at 540 nm (method A) and increase in the yellowish-orange color at 445 nm (method B) due to the bromination of the dye. Similar to method A, unreacted bromine would react with EGC and the decrease in the absorbance of the green color of EGC in acid medium was measured at 630 nm (method C). The tentative reaction scheme is given and illustrated in Figure 2.

### 3.3. Basis of the Methods

 ATN, when added in increasing concentrations to a fixed concentration of *in situ* bromine, consumed the latter and there occurred a concomitant fall in bromine concentration. When a fixed concentration of MCP was added to decreasing concentrations of bromine, a concomitant increase in the absorbance of MCP resulted at 540 nm and at the same time decrease in the absorbance resulted at 445 nm. Similarly, when a fixed concentration of EGC was added to decreasing concentrations of bromine, a corresponding increase in the absorbance of EGC was observed at 630 nm. These were observed as a proportional increase in the absorbance at 540 nm (method A) or 630 nm (method C) and decrease at 445 nm (method B) with increasing the concentration of ATN.

### 3.4. Optimization of Reaction Variables

#### 3.4.1. Effect of Reagent Concentration

 Preliminary experiments were performed to fix the upper limits of the MCP and EGC that could produce a reasonably high absorbance, and these were found to be 80, 200 *μ*g/mL for MCP in methods A and B, and 300 *μ*g/mL for EGC in method C. Bromate concentrations of 4.0 and 1.8 *μ*g/mL in the presence of excess bromide were found optimum to bleach the dye color in method A and method C, respectively, whereas 8.0 *μ*g/mL KBrO_3_ produced a reasonable maximum absorbance at 445 nm in method B. Hence, different concentrations of ATN were reacted with 1.0 mL each of 40, 80, and 18 *μ*g/mL bromate in methods A, B, and C, respectively.

#### 3.4.2. Effect of Reaction Medium

**Figure 3:**
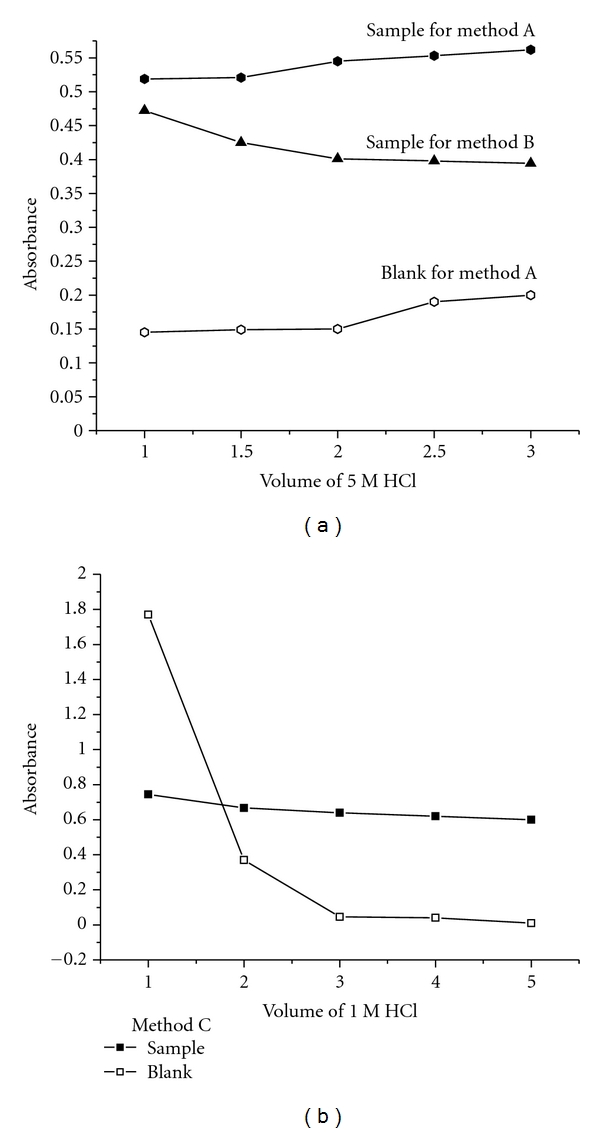
Effect of acid on the color development of the measured species for the proposed methods.

 Hydrochloric acid was found to be an ideal medium for the two steps involved in all the three methods (Figure 3). In method A, the effect of (1.0–3.0 mL of 5 M HCl) was studied and the results showed that 2.0 mL of 5 M HCl was optimum for the bromination reaction of the drug as well as the dye. Taking in to account the maximum absorbance of the measured species and the minimum absorbance of the blank, 2.0 mL of 5 M HCl was fixed. In method B, 2.0 mL of 5 M HCl was found optimum and any excess of the acid up to 3.0 mL would not affect the absorbance of the measured species. In method C, 5.0 mL of 1 M HCl was found optimum to achieve maximum absorbance for the sample and minimum absorbance for the blank.

#### 3.4.3. Reaction Time and Color Stability

The reaction time between ATN and the bromine generated *in situ* was found to be 15 min in method A and 10 min in both method B and method C. After completion the reaction between the drug and the bromine, the residual bromine would brominate the dyes and this bromination process was found to be complete in 5 min for all three methods. The absorbance of the measured species was constant up to 24 hours.

### 3.5. Validation of the Proposed Methods

#### 3.5.1. Linearity

 A linear relation is found between absorbance and concentration of ATN within Beer's law range given in Table 2. The calibration graphs are described by the equation:

(1) Y  =  a  +  b  X,

(where *Y* = absorbance, *a* = intercept, *b* = slope and *X* = concentration in *μ*g/mL) obtained by the method of least squares. The apparent molar absorptivity (*ε*), Sandell's sensitivity, limits of detection (LOD), and quantification (LOQ) are also given in the Table 2. Limits of detection (LOD) and quantification (LOQ) were calculated from the following equations [67]:

(2) $$\begin{array}{cc} \text{LOD} & =\frac{3.3\times \sigma }{S},\\ \text{LO}𝒬 & =\frac{10\times \sigma }{S}, \end{array}$$

where *σ* is the standard deviation of “*n*” reagent blank determinations and *S* is the slope of the calibration curve.

#### 3.5.2. Accuracy and Precision

 In order to study the precision and accuracy of the proposed methods, three concentrations of pure ATN within the linearity range were analyzed, each determination being repeated seven times (intraday precision) in the same day and one time each for five days (interday precision). The percentage relative standard deviation (%RSD) was ≤2.09% (intraday) and ≤2.63% (interday). In addition, the accuracy of the proposed method was measured by calculating the percentage relative error (%RE), which was varied between 0.46% and 3.54%. The results of this study indicate the high accuracy and precision of the proposed methods (Table 3).

#### 3.5.3. Robustness and Ruggedness

 To evaluate the robustness of the methods, two important experimental variables, namely, the amount of acid and reaction time, were slightly varied, and the capacity of the methods was found to remain unaffected by small deliberate variations. The results of this study are presented in Table 4 and indicate that the proposed methods are robust. Method ruggedness is expressed as %RSD of the same procedure applied by three analysts and using three different spectrophotometers by the same analyst. The interanalysts' and interinstruments' RSD values were ≤3.42% indicating ruggedness of the proposed methods. The results of this study are presented in Table 4.

#### 3.5.4. Selectivity

 In the present methods, a study of some potential interference was performed by selecting the excipients often used in pharmaceutical formulations or as possible coactive substances. Selectivity was evaluated by both placebo blank and synthetic mixture analyses. The placebo blank, consisting the composition as mentioned under Section 2.3.5 was prepared and analyzed as described under the recommended procedures. The resulting absorbance readings for the methods were the same as the reagent blank, inferring no interference from the placebo. The selectivity of the methods was further confirmed by carrying out recovery study from synthetic mixture. The percent recoveries of ATN were 102.1 ± 1.35, 101.9 ± 1.18, and 101.4 ± 1.63 for method A, method B, and method C, respectively. This confirms the selectivity of the proposed methods in the presence of the commonly employed tablet excipients.

#### 3.5.5. Application to Analysis of Pharmaceutical Samples

 The proposed methods were successfully applied to the determination of ATN in three different brands of tablets, namely, Atenex-25, Atekind-50, and Aten-100. The results presented in Table 5 showed that there was a close agreement between the results obtained by the proposed methods and the label claim. The results were also compared with those of the reference method [3] statistically by a Student's *t*- test for accuracy and variance ratio *F*-test for precision at 95% confidence level. The reference method consisted of the measurement of the absorbance of the methanolic tablet solution at 275 nm. The calculated *t*- and *F*-values indicate that there is no significant difference between the proposed methods and the reference method with respect to accuracy and precision.

#### 3.5.6. Recovery Studies

 To study the reliability of the proposed method, a standard addition technique was followed. A fixed amount of drug from preanalyzed tablet powder was taken and pure drug at three different concentrations (50, 100, and 150% of that in tablet powder) was added. The total concentration was found by the proposed methods. The determination with each concentration was repeated three times and the percent recovery of the added standard was calculated. Results of this study presented in Table 6 reveal that the accuracy of methods was unaffected by the various excipients present in the formulations.

## 4. Conclusions

Three sensitive spectrophotometric methods for the determination of ATN have been developed and validated based on the current ICH guidelines [67]. The present methods demonstrate that bromate-bromide mixture and metacresol purple or erioglaucine can be used for the quantitative determination of ATN in bulk drug as well as in tablets. The proposed methods have the advantages of utilization of bromine generated *in situ* as a green brominating reagent, free from critical experimental conditions, and complicated procedures such as heating or extraction step. The reagents used in the proposed methods are cheap, readily available, and the procedures do not involve any tedious sample preparation. These advantages encourage the application of the proposed methods in routine quality control analysis of ATN in pharmaceutical formulations.

## Figures and Tables

**Table 1:** Comparison of the proposed and the existing visible spectrophotometric methods.

Sl. No.	Reagent/s used	Reagent used	*λ*_max⁡_ (nm)	Linear range, *μ*g/mL and **ε**, L/mol · cm	LOD, *μ*g/mL	Reaction time, min	Remarks	Reference
(1)	Hydroxylamine hydrochloride-iron (III)	Ferric hydroxamate complex measured	510	50–800 (*ε* = 5.3 × 10^2^)	NR	20–30	Less sensitive, heating required	[51]

(2)	Chloranilic acid	Charge transfer complex measured	534	25–250	NR	—	Less sensitive, use of organic solvents	[52]

(3)	Chloranilic acid	Charge transfer complex measured	530	10–280	NA	NA	-do-	[53]

(4)	Acetaldehyde-Chloranil		690	NA	NA	NA	Use of organic solvents	[54]

(5)	NH_4_VO_3_	Reaction rate measured	750	NA	NA	NA	Heating required	[55]

(6)	4-chloro-7-nitrobenzo-2-oxa-1,3-diazole	Coupling product measured as a function of time	460	5–50	1.3	30	Heating required	[56]

(7)	Potassium permanganate- in alkaline medium	Unreacted KMnO_4_ measured	526			4 hrs	Time-consuming, involve judicial control of many experimental variables	[57]
		Rate-constant method		6.66–10.65				
		Fixed-concentration method		6.66–5.33				
		Fixed-time method		6.66–7.99				

(8)	Sodium nitroprusside	Complex of ammonia and nitroprusside measured	495	0.5–30 (*ε* = 3.01 × 10^5^)	0.01	5	Heating required	[58]

(9)	Chloramine-T-metol-sulphanilic acid	Unreacted chloramine-T measured	520	2.5–25 (*ε* = 3.24 × 10^3^)	2.34	20	Less sensitive	[59]

(10)	Chloramine-T:							
	(a) Metanil yellow	Unreacted chloramine-T measured	530	1–12 (*ε* = 1.19 × 10^4^)	0.32	10		[60]
	(b) Indigo carmine		610	2.5–20 (*ε* = 6.65 × 10^3^)	0.04	10		

(11)	Bromate-bromide mixture- methyl orange	Unreacted bromine measured	520	0.5–4.0 (*ε* = 4.13 × 10^4^)	0.07	15		[61]

(12)	Phenol red	The change in the color of phenol red measured	430	3.0–30 (*ε* = 3.47 × 10^3^)	4.61	—	Less sensitive	[62]

(13)	Bromate-bromide mixture:						Simple, sensitive and no heating step. No use of organic solvent. Use of an eco-friendly brominating reagent.	This work
	(a) MCP	Unreacted MCP in acid measured	540	1.0–20.0 (*ε* = 1.20 × 10^4^)	0.12	15		
	(b) MCP	Bromo-derivative of MCP measured	445	2.0–40.0 (*ε* = 4.51 × 10^3^)	0.56	10		
	(c) EGC	Unreacted EGC in acid measured	630	1.0–8.0 (*ε* = 3.46 × 10^4^)	0.05	10		

MCP: metacresol purple, EGC: erioglaucine, NR: not reported, NA: not available.

**Table 2:** Regression and analytical parameters.

Parameter	Method A	Method B	Method C
*λ*_max⁡_, nm	540	445	630
Beer's law limits (*μ*g/mL)	1–20	2–40	1–8
Molar absorptivity (L/mol · cm)	1.20 × 10^4^	4.51 × 10^3^	3.46 × 10^4^
Sandell sensitivity* (*μ*g/cm^2^)	0.0223	0.0591	0.0077
Limit of detection (*μ*g/mL)	0.12	0.56	0.05
Limit of quantification (*μ*g/mL)	0.36	1.69	0.14
Regression equation, Y**			
Intercept	0.0038	0.7755	0.0217
Slope	0.0443	−0.0154	0.1229
Correlation coefficient (*r*)	0.9996	−0.9973	0.9992
Standard deviation of intercept (*S*_*a*_)	0.00664	0.08471	0.01436
Standard deviation of slope (*S*_*b*_)	0.00059	0.00378	0.00292

*Limit of determination as the weight in *μ*g per ml of solution, which corresponds to an absorbance of *A* = 0.001 measured in a cuvette of cross-sectional area 1 cm^2^ and l = 1 cm. **Y = *a* + *bX*, where *Y* is the absorbance, *a* is the intercept, *b* is the slope, and *X* is the concentration in *μ*g/mL.

**Table 3:** Evaluation of intraday and interday precision and accuracy.

Method	ATN taken (*μ*g/mL)	Intraday (*n* = 7)			Interday (*n* = 5)		
		ATN found^a^ (*μ*g/mL )	%RSD^b^	%RE^c^	ATN found^a^ (*μ*g/mL )	%RSD^b^	%RE^c^
Method A	4.00	4.14	1.49	1.71	4.09	1.86	2.25
	8.00	8.12	0.75	1.56	8.16	1.34	2.00
	12.00	4.0	0.67	1.04	12.31	1.28	2.58
Method B	8.00	8.22	1.74	2.69	8.19	2.14	2.38
	16.00	16.25	1.06	1.58	16.44	2.08	2.75
	24.00	24.63	0.56	2.62	24.85	1.72	3.54
Method C	2.00	1.99	1.64	0.46	2.05	2.14	2.50
	4.00	4.09	2.09	2.44	4.14	2.56	3.50
	6.00	6.07	1.47	1.09	6.16	2.63	2.67

^a^Mean value of five determinations;   ^b^relative standard deviation (%);  ^c^relative error (%).

**Table 4:** Robustness and ruggedness.

Method	ATN taken, *μ*g/mL	Method robustness		Method ruggedness	
		Parameters altered			
		Volume of acid, ml^a^ RSD, % (*n* = 3)	Reaction time^b^ RSD, % (*n* = 3)	Interanalysts' RSD, % (*n* = 3)	Inter instruments' RSD, % (*n* = 3)
A	4.00	1.26	1.46	1.34	2.64
	8.00	0.72	1.72	0.85	3.18
	12.00	0.64	1.28	1.03	3.03
B	8.00	0.85	1.39	1.42	2.86
	16.00	0.52	0.92	1.17	2.47
	24.00	1.18	1.15	1.33	3.26
C	2.00	1.26	1.26	1.06	3.42
	4.00	0.96	1.39	0.88	2.78
	6.00	1.08	0.76	1.24	2.37

^a^In methods A and B, the volume of 5 M HCl was 1.8, 2.0, and 2.2 mL whereas in method C, the volume of 1 M HCl was 4.8, 5.0, and 5.2 mL ^b^ The reaction time in methods A was 14, 15, and 16 min whereas in methods B and C, the same was 9, 10, and 11 min.

**Table 5:** Results of analysis of tablets by the reference and proposed methods.

Tablet Brand name	Label claim mg/tablet	Found (percent of label claim ± SD)^a^			
		Reference method	Proposed methods		
			Method A	Method B	Method C
Atenex-25^b^	25	100.3 ± 0.58	100.9 ± 1.06	99.65 ± 0.96	101.0 ± 1.12
			*t* = 1.11	*t* = 1.2	*t* = 1.28
			*F* = 3.34	*F* = 2.74	*F* = 3.33
Atekind-50^c^	50	99.67 ± 0.67	101.0 ± 1.09	100.6 ± 1.36	99.81 ± 1.42
			*t* = 2.32	*t* = 1.42	*t* = 0.20
			*F* = 1.48	*F* = 4.12	*F* = 4.49
Aten-100^d^	100	100.6 ± 0.82	100.6 ± 1.11	101.1 ± 1.37	99.72 ± 1.69
			*t* = 0.03	*t* = 0.69	*t* = 1.05
			*F* = 1.83	*F* = 2.79	*F* = 4.25

^a^Mean value of five determinations. ^ b,d ^Marketed by Zydas Healthcare, East Sikkim, India, ^c^ Marketed by Mankind Pharma Ltd., New Delhi, India, Tabulated *t*-value at the 95% confidence level is 2.78. Tabulated *F-*value at the 95% confidence level is 6.39.

**Table 6:** Results of recovery study by standard addition method.

Tablets studied	Method A				Method B				Method C			
	ATN in tablets, *μ*g/mL	Pure ATN added, *μ*g/mL	Total found, *μ*g/mL	Pure ATN recovered*, percent ± SD	ATN in tablets, *μ*g/mL	Pure ATN added, *μ*g/mL	Total found, *μ*g/mL	Pure ATN recovered*, percent ± SD	ATN in tablets, *μ*g/mL	Pure ATN added, *μ*g/mL	Total found, *μ*g/mL	Pure ATN recovered*, percent ± SD
Atenex 25	4.08	2.0	6.07	99.5 ± 2.29	7.97	4.0	12.01	101.00 ± 2.74	2.02	1.0	3.05	103.00 ± 1.76
	4.08	4.0	8.03	98.75 ± 2.79	7.97	8.0	15.93	99.50 ± 2.35	2.02	2.0	4.09	103.5 ± 1.98
	4.08	6.0	10.15	101.17 ± 1.22	7.97	12.0	20.10	101.08 ± 0.98	2.02	3.0	5.11	103.00 ± 1.52
Atekind 50	3.99	2.0	6.06	103.5 ± 1.91	8.05	4.0	12.16	102.75 ± 2.15	1.99	1.0	3.00	101.00 ± 0.97
	3.99	4.0	8.14	103.75 ± 1.04	8.05	8.0	16.15	101.25 ± 2.76	1.99	2.0	4.02	101.50 ± 1.58
	3.99	6.0	10.23	104.00 ± 2.51	8.05	12.0	20.44	103.25 ± 2.40	1.99	3.0	5.03	101.33 ± 1.79
Aten 100	4.02	2.0	6.10	104.00 ± 2.31	8.09	4.0	12.18	102.25 ± 1.56	1.99	1.0	3.02	103.00 ± 2.13
	4.02	4.0	8.16	103.5 ± 1.43	8.09	8.0	16.35	103.25 ± 2.45	1.99	2.0	4.05	103.00 ± 2.06
	4.02	6.0	10.29	104.5 ± 2.77	8.09	12.0	20.34	102.08 ± 1.47	1.99	3.0	5.07	102.67 ± 1.91

*Mean value of three determinations.
